# Broad brain networks support curiosity-motivated incidental learning of naturalistic dynamic stimuli with and without monetary incentives

**DOI:** 10.1162/imag_a_00134

**Published:** 2024-05-01

**Authors:** Stefanie Meliss, Aki Tsuchiyagaito, Phoenix Byrne, Carien van Reekum, Kou Murayama

**Affiliations:** School of Psychology and Clinical Language Sciences, University of Reading, Reading, United Kingdom; Ambition Institute, London, United Kingdom; Laureate Institute for Brain Research, Tulsa, OK, United States; Oxley College of Health Sciences, The University of Tulsa, Tulsa, OK, United States; Research Center for Child Mental Development, Chiba University, Chiba, Japan; Hector Research Institute of Education Sciences and Psychology, Tübingen, Germany

**Keywords:** curiosity, encoding, fMRI, naturalistic, intersubject correlation, magic tricks

## Abstract

Curiosity—the intrinsic desire to know—is a concept central to thehuman mind and knowledge acquisition. Experimental studies oninformation-seeking have found that curiosity facilitates memory encoding andexhibits similar rewarding properties as extrinsic rewards/incentives, byeliciting a dopaminergic response in the reward network. However, it is notclear whether these findings hold with more naturalistic dynamic stimuli and howthe joint effect of curiosity and extrinsic incentive manifests in learning andneural activation patterns. Herein, we presented participants with videos ofmagic tricks across two behavioural (N_1_= 77, N_2_= 78) and one fMRI study (N = 50) and asked them to ratesubjective feelings of curiosity, while also performing a judgement task thatwas incentivised for the half of participants. Incidental memory for the magictrick was tested a week later. The integrated results showed that both curiosityand availability of extrinsic incentives enhanced encoding but did not interactwith each other. However, curiosity influenced only high-confidence recognitionmemory, whereas extrinsic incentives affected memory regardless of confidence,suggesting the involvement of different encoding mechanisms. Analysis of thefMRI data using the intersubject synchronisation framework showed that, whilethe effects of curiosity on memory were located in the hippocampus anddopaminergic brain areas, neither the effects of curiosity nor incentivesthemselves were found in the often-implicated reward network. Instead, they wereassociated with cortical areas involved in processing uncertainly and attention.These results challenge a traditional focus on reward networks in curiosity andhighlight the involvement of broader brain networks.

## 1 Introduction

“We find ourselves in a bewildering world. We want to make sense of whatwe see around us and to ask: what is the nature of the universe? What is ourplace in it and where did it and we come from? Why is it the way itis?”

( Hawking, 2016 , p. 205)

With the above words, Stephen Hawking introduced the concluding chapter of his famousbook “A Brief History Of Time”, where he aimed to explain our universeto a non-scientific audience. The wonder the words capture, the intrinsic desire toknow, has not only motivated scientists to dedicate their careers to trying to findanswers to the big questions of the universe, but also the readers of the more than10 million copies sold to spend their time and monetary resources to acquireknowledge about the Big Bang. This is intriguing because, arguably for most of them,being able to understand how to combine weak and strong nuclear forces with those ofgravity and electromagnetism into a single unified theory will have no instrumentalvalue to maximise their rewards in their everyday lives.

In line with this anecdotal evidence, research has found that humans actively engagein non-instrumental information-seeking (Kobayashiet al., 2019;van Lieshout et al.,2021), even if it requires a small cost (Bennett et al., 2016;Brydevall et al.,2018;Kang et al., 2009;Kobayashi & Hsu, 2019;Marvin & Shohamy, 2016;van Lieshout et al., 2018), involves taking the risk ofreceiving an electric shock (Lau et al.,2020), or leads to experiencing negative emotions like regret (FitzGibbon et al., 2021). These observationshave led researchers to propose that information is a reward (FitzGibbon et al., 2020;Marvin & Shohamy, 2016), functioning like extrinsic rewards(e.g., food or money) to govern our behaviour (Murayama, 2022;Murayama et al.,2019). In fact, in monkeys, the same dopaminergic neurons in the midbrainthat signal the expected amount of primary extrinsic rewards also signal theexpectation of information (Bromberg-Martin &Hikosaka, 2009). Likewise, in humans, the subjective value of informationand basic extrinsic rewards share a common neural code expressed in the striatum andother reward-related areas, such as the ventromedial prefrontal cortex (Kang et al., 2009;Kobayashi & Hsu, 2019;Lau et al., 2020).

### 1.1 Curiosity-motivated learning

The subjective feelings underlying our desire to know—which we will referto as the subjective feeling of*curiosity*—have beenshown to facilitate memory encoding (for recent reviews, seeGruber & Ranganath, 2019;Gruber et al., 2019). More specifically, thesubjective feeling of curiosity elicited by a curiosity-triggering cue (i.e., atrivia question; cf.Jepma et al., 2012)facilitates the intentional encoding (Duan etal., 2020;Halamish et al.,2019) of the target item (i.e., the answer to the trivia question;cf.Jepma et al., 2012). The samecuriosity effects have also been found in incidental encoding paradigms aftershort (Brod & Breitwieser, 2019;Galli et al., 2018;Gruber et al., 2014;Jepma et al., 2012;Ligneul et al.,2018;Murphy, Dehmelt, et al.,2021;Poh et al., 2021;Mullaney et al., 2014;Stare et al., 2018) and long (Fastrich et al., 2018;Gruber et al., 2014;Kang et al., 2009;Marvin &Shohamy, 2016;Murayama &Kuhbandner, 2011;Stare et al.,2018;Swirsky et al., 2021)intervals. Interestingly, incidental information, which is semanticallyunrelated to the cue eliciting the feeling of curiosity but presented in closetemporal proximity (i.e., during a state of high compared to low curiosity), isalso preferentially encoded (Galli et al.,2018;Gruber et al., 2014;Murphy, Dehmelt, et al., 2021;Stare et al., 2018).

Neuroimaging research has suggested that such curiosity-motivated learning isrelated to the activity and interaction between three brain areas: the nucleusaccumbens (NAcc), the dopaminergic midbrain (VTA/SN), and the hippocampus (HPC).Specifically,Gruber and colleagues(2014)investigated whether brain activity during curiosityelicitation at cue presentation (i.e., the trivia question) predicts latermemory for the upcoming target information (i.e., the answer to the triviaquestion). They found that while the dopaminergic midbrain was more activatedduring the anticipation of later remembered, relative to later forgottentargets, irrespective of the degree of curiosity elicitation, the right HPC andthe bilateral NAcc showed increased activation for remembered as opposed toforgotten targets, specifically for high-curiosity cures. They also found astrong correlation between the curiosity-driven memory benefit for incidentalinformation and the curiosity-related subsequent memory effects in the VTA/SNand the HPC. Increased functional connectivity between them was evidentparticularly in high, but not in low curiosity trials. Taken together, theresults suggest that anticipatory activity in the mesolimbic dopaminergiccircuit and the HPC supports the learning benefits associated with high comparedto low states of curiosity (Gruber &Ranganath, 2019).

However, despite the increasing amount of research on curiosity-motivatedlearning, the vast majority of studies have relied only on a single type ofmaterial (for exceptions, see e.g.,Cen et al.,2021;Jepma et al.,2012)—trivia questions (e.g.,Fastrich et al., 2018;Gruber etal., 2014;Kang et al., 2009;Marvin & Shohamy, 2016;Murayama & Kuhbandner, 2011;Wade & Kidd, 2019). Trivia questionparadigms, while advantageous for studying curiosity, has a notable limitation:it primarily examines a type of curiosity triggered by the detection of a gap inone’s knowledge (i.e., information-based prediction errors;Gruber & Ranganath, 2019). While aknowledge gap can provoke the feeling of curiosity (Loewenstein, 1994), there has been increasingconsensus that curiosity can stem from various sources, each potentiallyinvolving different psychological and neural mechanisms (Gruber & Ranganath, 2019;Jach et al., 2022;Kobayashi et al., 2019;Sharot& Sunstein, 2020). In fact, curiosity is not just aboutfilling knowledge gaps; the subjective feeling of curiosity can be elicited innovel environments or through events that violate our expectations, creating asense of surprise (i.e., context-based prediction errors;Gruber & Ranganath, 2019). The violation ofexpectations has been shown to stimulate surprise and curiosity, and tofacilitate learning (Brod & Breitwieser,2019;Brod et al., 2018); andis considered a reliable predictor of curiosity (Vogl et al., 2019). More so,Ligneul and colleagues (2018)showed that surprisemediated the effects of curiosity on memory, with higher surprise levels leadingto more activation in the ventromedial prefrontal cortex (vmPFC), andsubsequently, better memory for certain items. Despite such intriguingpreliminary findings, the role of this surprise-based curiosity effect on memoryencoding and its neural underpinnings remain under-examined.

To examine surprise-based curiosity, the current study used novel naturalisticstimuli that strongly trigger that type of curiosity—videos of magictricks. Magic tricks induce curiosity independent of language and priorknowledge by showing implausible or impossible events (Kuhn et al., 2008;Rensink & Kuhn, 2014). Importantly, magic trick videoclipssubsume a sequence of events that typically triggers surprise-based curiosity:the formation of expectation, the violation of these expectations, a subjectivefeeling and experience of curiosity, and finally, internal search for potentialexplanations (e.g., “how could that be possible?”). Magic tricksare created specifically to induce feelings of surprise: Magicians purposefullyproduce a sequence of dynamic events that orient the viewer’s predictionsin a certain direction, only to then present events that violate these formedpredictions. This technique sequence makes magic tricks an effective tool foreliciting a strong form of surprise-based curiosity, known as context-based orperceptual prediction error (Zacks et al.,2007)^1^. Indeed, previous research hasshown that magic tricks are perceived as surprising, and violate cause andeffect relations, leading to unexpected outcomes (Danek et al., 2015;Parris et al., 2009). Furthermore, they trigger epistemic emotions(surprise in response to the trick, interest in the trick, and curiosity aboutthe solution;Ozono et al., 2021), andeven elicit curiosity-driven risky decision-making, akin to the effects oftrivia questions, supported by activation in the ventral striatum (Lau et al., 2020). Therefore, magic trickscan be considered as one of the most suitable class of stimuli to understand theneural processes underlying surprise-based curiosity.

### 1.2 Role of extrinsic incentives

Another critical issue is the role of extrinsic incentives and rewards^2^in curiosity-motivatedlearning. Overall, the facilitating effects of curiosity on memory encoding beara striking resemblance to the effects of extrinsic rewards on memory in theliterature (for a review, seeMiendlarzewska etal., 2016): it has been shown that providing monetary incentives andrewards not only increases intentional encoding of incentivised items (Adcock et al., 2006;Gruber & Otten, 2010;Gruber et al., 2013;Wolosin et al., 2012), but also the incidental encoding ofinformation presented in the context of a rewarded task (Bunzeck et al., 2010,^2012^;Gruber et al., 2016;Murayama & Kitagami, 2014;Murty & Adcock, 2014;Patil et al., 2017;Stanek et al., 2019;Wittmann et al., 2005,^2008^,^2011^). Neuroimaging studies havelinked this behavioural incentive effect on intentional encoding to activity inNAcc, HPC and VTA, showing an enhanced activity during cue presentation forlater remembered compared to forgotten targets, only in the context of high, butnot low rewards (Adcock et al., 2006).Furthermore, they showed that functional connectivity between HPC and VTA/SNsupports the behavioural reward effect (Adcock etal., 2006;Wolosin et al.,2012). This involvement of VTA/SN and HPC is consistent with thehypothesis that reward promotes memory formation, via dopamine releasemodulating hippocampal synaptic encoding processes during long-term potentiation(Lisman & Grace, 2005;Lisman et al., 2011;Shohamy & Adcock, 2010).

While the effects of monetary incentives/rewards and, more recently, curiosityhave been studied in isolation leading to valuable insights, only a smallportion of studies have actually looked at them in conjunction. Studying botheffects in the same study is necessary to closely understand the similaritiesand differences of neural mechanisms in how they benefit learning. Murayama andKuhbander (2011) found that both monetary reward and the interestingness oftrivia questions, as rated by a separate sample, had an enhancing effect onencoding. However, the main effects were further qualified by an interaction,where monetary rewards only enhanced encoding of trivia questions rated as notinteresting. The findings were replicated in younger and older adults (Swirsky et al., 2021), although some otherstudies failed to find the interaction effects (Duan et al., 2020;Halamish et al., 2019). Thus, theliterature suggests the possibility that there may be unique non-additive neuralpatterns when both curiosity and monetary incentives are present.

### 1.3 Current research

The current study aims to examine curiosity-motivated learning, with a specificfocus on surprise-based curiosity. To achieve this objective, we utilised videosof magic tricks to induce curiosity, and examined the neural dynamics underlyingcuriosity-triggering processes, starting from the initial formation ofexpectations to the subsequent search for potential explanations in thepost-effect phase. Additionally, we manipulate the availability of extrinsicincentives in our study design. This allows us to examine the potentialinteractive effects of curiosity and extrinsic incentives on learning. Asindicated earlier, despite the strong suggestion that information-seeking isdriven by reward learning, neuroimaging studies on motivated learning examinedcuriosity and extrinsic incentives somewhat individually, making it difficult tounderstand how these two types of motivating factors enhance (or do not enhance)memory in tandem. The current study provides a first attempt to examine theinteractive effect using fMRI.

We conducted three studies (two behavioural and one using fMRI), all sharing asimilar structure. In each experiment, participants viewed a series of magictrick videos and performed a judgement task including curiosity ratings. Toexamine the effects of extrinsic incentives, half of the participants werepromised additional monetary bonus payments for the judgement task, whereas theremaining half of participants did not receive such an incentive. A week later,memory for the magic tricks was assessed using surprise recognition and recalltests. Based on the previous literature, we hypothesised that both curiosity andmonetary incentives would facilitate memory encoding, both of which may besupported by similar neural processes located in the hippocampal-VTA loop (Lisman & Grace, 2005). We alsoexpected an interaction between curiosity and monetary incentives, both onbehavioural measures of memory and the neural activation in the hippocampal-VTAloop, to show the positive effect of extrinsic incentives, of which may onlymanifest for less curious magic tricks

## 2 Methods

### 2.1 Study 1: Behavioural study

#### 2.1.1 Participants & design

The*a priori*defined intended sample size was a total 80participants. This was mainly limited by the budget, but our sensitivityanalysis showed that this sample size is sufficient to detect medium-sizedeffects, at 80% of power for the between-subjects effect of monetaryincentives (*d*= 0.63). Given that the reward effectson memory have been established in the literature (Adcock et al., 2006;Gruber et al., 2016;Miendlarzewska et al., 2016;Wittmann et al., 2005), we decided to go withthis sample size. Participants were recruited using Prolific (https://prolific.co) for anonline study consisting of two parts, spaced 1 week apart. Both parts tookapproximately 45 min each, with a participant time reimbursement totalling£7.50. For inclusion, the following criteria were defined: agebetween 18 and 37, fluency in English, a minimum approval rate of 95%, andat least 10 previous submissions.

Unbeknown to the participants, the study included a between-group incentivemanipulation, where the experimental group was instructed that they couldearn additional monetary bonus payments for their performance in thejudgement task, whereas the control group did not receive such instructions.The bonus amount was defined as £0.10 per correct answer in thejudgement task. By incentivising performance in the judgement task, ratherthan in the memory assessment, our task examines the effects of monetaryincentives on incidental encoding.

Considering potential attrition, we oversampled participants against thepredefined sample size. In total, we received data from 47 and 44participants in the control and incentives condition, respectively, out ofwhich five and three participants were excluded due to incomplete data. All83 participants who had submitted complete datasets were invited toparticipate in the second part of the study. Of this sample, 42 participantsfrom the control and 39 participants from the incentive group responded. Intotal, four datasets were excluded from the second part (3 due to incompletedata and 1 due to a self-reported age below 18, all from the controlcondition). The final sample size included in the analysis included N= 77 participants (n_control_= 38,n_incentive_= 39). The participant characteristics aredescribed inTable 1. The study wasreviewed and approved by the University of Reading’s School ResearchEthics Committee (SREC; 2016-109-KM).

**Table 1.** Participant characteristics.

	Behavioural study		Replication		fMRI study	
	Controlgroup	Incentivegroup	Controlgroup	Incentivegroup	Controlgroup	Incentivegroup
Subjects per group	n = 38	n = 39	n = 40	n = 38	n = 25	n = 25
Age	27.87 (4.58)[18; 35]	26.46 (5.14)[18; 35]	25.62 (4.89)[18; 35]	26.24 (4.70)[18; 35]	26.52 (5.46)[18; 37]	24.12 (4.70)[19; 37]
Gender (% female)	36.84	38.46	30.00	39.47	68.00	76.00
Ethnicity (% BAME)	60.53	66.67	60.00	39.47	32.00	24.00
Years of Education	14.46 (1.77)[10; 18]	14.72 (2.99)[8; 24]	13.43 (2.72)[5; 17]	15.08 (1.89)[12; 21]	16.12 (2.62)[13; 22]	15.92 (2.04)[11; 19]
Days between sessions	7.21 (0.66)[6.90; 10.80]	7.22 (0.50)[6.90; 8.99]	7.29 (1.02)[6.29; 10.82]	7.23 (0.82)[6.57; 11.02]	7.50 (0.67)[6.83; 9.49]	7.36 (0.45)[6.87; 8.20]
Experience with magic	1.66 (0.99)[1.00; 4.00]	1.36 (0.71)[1.00; 4.00]	1.68 (0.89)[1.00; 4.00]	1.76 (0.75)[1.00; 3.00]	1.56 (0.87)[1.00; 4.00]	1.44 (0.77)[1.00; 4.00]

*Note*. For interval-scaled variables, the tableshows the mean (standard deviation) [minimum; maximum]separately for each group and data collection. Experience withmagic tricks relates to the participant’s rating of theirexperience in producing magic tricks on a scale from 1 =“never” to 6 = “veryfrequently”.

#### 2.1.2 Material

We displayed short magic trick videos to participants. The magic trick videoswere selected from the Magic Curiosity Arousing Tricks (MagicCATs) stimuluscollection (Ozono et al., 2021). Thiscollection was developed specifically for fMRI experiments, containing 166magic tricks that were edited to achieve a similar background and viewingfocus, and muted purposefully to minimise the effects of verbalinterference. To select magic tricks used here, the following criteria wereapplied: (1) duration between 20 and 60 s, (2) broad range of differentmaterials and features so that magic tricks are distinguishable in a cuedrecall paradigm, (3) varying degrees of curiosity ratings as reported in thedatabase, and (4) understandable without the use of subtitles. Additionalediting was performed using Adobe^®^Premiere ProCC^®^(2015) software where needed, for instance, toremove subtitles. Magic tricks were exported in a slightly larger size thanavailable in the database (1280 x 720 pixels). In total, 36 magic trickswere displayed in the experiment and an additional two were used forpractice trials. This number is equivalent to what has been used previouslywhen studying decision-making using magic tricks (Lau et al., 2020). Average memory performance orcuriosity ratings were not significantly correlated with video length oraverage frame-by-frame luminance (*ps*> .12). PleaseseeMeliss et al. (2022)for moreinformation about the magic tricks used.

A frame of each magic trick video was extracted as a cue image (1920 x 1080pixels) for the memory test. For this, a frame was selected from before themoment(s) of surprise (i.e., moments violating one’s expectations)that was distinctive enough to cue the magic trick without revealing itentirely.

#### 2.1.3 Tasks & measurements

##### 2.1.3.1 Magic trick watching task

During each trial of the magic trick watching task (seeFig. 1, upper half), participantswatched a magic trick video and were then asked to estimate how manypeople (out of 100) are able to correctly figure out the solution. Forthis, participants could choose out of the following answer options:“0–10%”, “11–20%”,“21–30 %”, and “31 % and more”.Afterwards, participants were asked to rate how curious they were whilewatching the magic trick on a 7-point Likert scale (1 =“not curious at all”, 7 = “verycurious”). Importantly, the estimate rating was included tomanipulate incentives between subjects. The incentive manipulation waspart of the task instructions, which is described below.

**Fig. 1.**
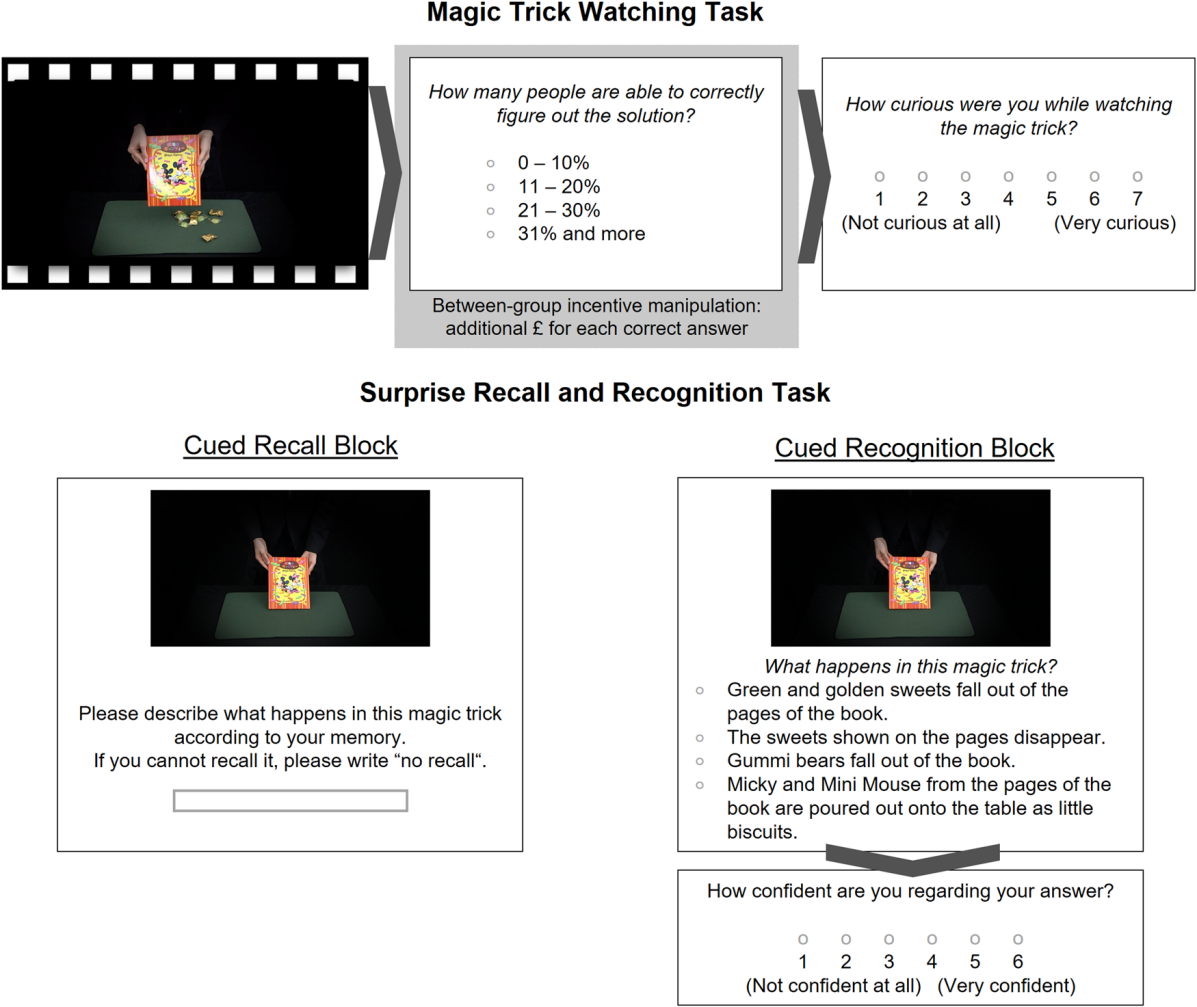
Overview of the task trials.*Note*. The figureillustrates the incidental incentives-motivated learning task aswell as the surprise memory test. Task flow is indicated usingdark grey arrows. The upper half of the picture shows the magictrick watching task trial as used in online studies. After amagic trick was displayed, participants were asked to give anestimate of how many people (out of 100) could find the solutionto the magic trick. In a between-subject design, participantswere instructed that they could earn additional monetary rewardsfor each correct estimate or did not receive such aninstruction. Participants were further asked to rate theircuriosity regarding the magic trick. The same task was used inthe fMRI experiment, but stimuli were edited and jitteredfixations in between the magic trick video and ratings wereintroduced. For more details, please refer to the taskdescription below or seeMelisset al. (2022). The lower half shows the memory taskconsisting of a cued recall and cued recognition block. Cueimages were taken from the magic tricks and the same images wereused during both blocks.

In total, the magic trick watching task consisted of 36 trials randomisedacross three blocks (12 trials each). There were no time-fixed responsewindows. Participants were able to take breaks in between blocks(self-paced).

##### 2.1.3.2 Surprise recall and recognition task

Approximately 1 week later, participants’ memory for the magictricks was tested using a surprise cued recall and a four-alternativeforced-choice recognition block (seeFig.1, lower half). During each trial in the cued recall block,the cue image was presented, and participants were asked to describewhat has happened in the cued magic trick according to their memoryusing a free answer format text input. They were instructed to be asdescriptive and detailed as possible because their answers would be usedto categorise whether they remembered a magic trick. Additionally, theywere asked to write “no recall” if they were unable torecall what happened.

During the cued recognition task trials, the same cue image waspresented, but this time paired with four choices to answer the questionof what happened in this magic trick. The answer options were presentedin random order. Behavioural piloting was conducted to achieve wordingsof distractor items that do not lead to floor or ceiling effects. Afterparticipants selected an answer (self-paced), they were asked to ratetheir confidence on a scale from 1 (“not confident atall”) to 6 (“very confident”). All 36 magic trickswere cued in the recall and recognition task in independent, randomorder. A break was offered in between both blocks.

##### 2.1.3.3 Task motivation inventory (TMI)

To measure task-dependent motivational constructs after the magic trickwatching task, the Task Motivation Inventory (TMI) was used. Morespecifically, the subscales intrinsic motivation (3 items;Elliot & Harackiewicz,1996), task engagement (3 items;Elliot & Harackiewicz, 1996), interest (3 items;Wigfield & Eccles,2000), boredom (3 items;Pekrun et al., 2002), effort (5 items;Ryan, 1982), and pressure (5 items;Ryan, 1982)^3^were used. Participantsanswered on a 7-point Likert scale from 1 (“definitelydisagree”) to 7 (“definitely agree”). The itemorder was randomised, but the same order was used across allparticipants.

#### 2.1.4 Experimental procedure

Participants were informed prior to starting the first part that they will beinvited to a second part. They were asked to only proceed with the firstpart if they could participate in the second part 1 week later. Afterproviding informed consent, participants filled in a demographicsquestionnaire. Afterwards, participants read through the task instructionscontaining the between-subject incentive manipulation. Half of theparticipants (incentives condition) were instructed that they could earn anadditional £0.10 for each time they estimated correctly how manypeople would be able to figure out the solution to the magic trick(pseudo-task). The other half of the participants, however, did not receivesuch an instruction (control condition). Participants were additionallyinformed that another study was run simultaneously on Prolific, indicatingthat there was a correct estimate, but that the data collection was stillrunning so there was no feedback. Afterwards, participants completed 2practice trials followed by 36 trials of the magic trick watching taskdistributed across three blocks. At the end, participants completed the TMI.A week later, participants were invited to participate in the second part ofthe study consisting of the surprise recall and recognition task. Bothexperiments were executed using a developmental version of Collector (Haffey et al., 2020)

### 2.2 Study 2: Replication behavioural study

#### 2.2.1 Participants & design

To ensure the robustness of effects, we ran a replication of the initialbehavioural study with small adjustments. The study was again conductedusing Prolific aiming for the predetermined sample size of 40 participantsper group applying the same inclusion criteria. Akin to the initialbehavioural study, the replication study was set up as a two-part study,spaced 1 week apart. The incentive manipulation was operationalised using abetween-subject design, with a set-up of two different studies on Prolific.The wording of the incentive manipulation was adopted so that it could betranslated to other study settings. More specifically, participants in theincentives condition were informed of the possibility to earn an additional50% bonus payment, on top of the payment for both tasks, if they estimatedcorrectly how many people would be able to figure out the solution.Participants were told that this would translate to an additional£0.10 per correct estimate. Participants were reimbursed £7.50for their time and received a bonus payment of £0.90 upon completingboth parts, mirroring chance-level performance in the pseudo-task.

Complete data from the first session were received from 40 participants ineach group. Due to 2 participants in the incentive group not completing thesecond session, the final sample size included in the analysis was N= 78 participants (n_control_= 40,n_incentive_= 38). The sample description can be foundinTable 1. The study was conductedas part of the same ethics approval mentioned above (2016-109-KM).

#### 2.2.2 Material

The same magic trick movie stimuli and cue images were used as describedabove.

#### 2.2.3 Tasks & measurements

The same tasks as described above were used. Small adjustments were made inthe wordings in the recognition task items to enhance readability (e.g., byadding articles). Additionally, the TMI included all five items for thepressure scale.

#### 2.2.4 Experimental procedure

Procedures were not modified in between data collections other than theabove-mentioned change in the wording of the incentive manipulation. Datawere collected using a later developmental version of Collector.

### 2.3 Study 3: fMRI study

In addition to behavioural effects, we were also interested in the neuralmechanisms underlying curiosity-motivated learning of dynamic stimuli;therefore, we adapted the magic trick watching task for use in the fMRI scanner,while adding a 10 min rest pre- and post-learning (data not included here). Thewhole MRI dataset has been made publicly available as the Magic, Memory, andCuriosity (MMC) Dataset (https://doi.org/10.18112/openneuro.ds004182.v1.0.0) and the task datawere analysed for this report. We here briefly summarise the methods, while amore detailed description can be found elsewhere (Meliss et al., 2022).

#### 2.3.1 Participants & design

Participants (seeTable 1fordemographic information) were recruited using leaflets that were distributedaround the campus to achieve a final sample size of N = 50 (i.e., 25participants per group). Participants were required to be right-handed. The*a priori*sample size considerations were based onsample sizes used in previous behavioural studies (Murayama & Kuhbandner, 2011), as well ason sample size recommendations for between-subject effects in naturalisticimaging (Pajula & Tohka, 2016;Yeshurun et al., 2017). It isimportant to note that the current study focuses on intersubject correlationanalysis, where pairs of participants are treated as the unit of analysis.This means that we have a larger sample size for statistical analysis. Whilewe correct for the dependence of these pairs (using mixed-effects models),normally we still have much higher statistical power than the analysis usingparticipants as the unit of analysis (McNabbet al., 2020). Similar to the behavioural studies, the fMRI studyconsisted of multiple sessions: a pre-scanning online assessment, the fMRIlab experiment where the magic trick task was performed inside the MRIscanner, and the surprise memory session performed online a week later. Intotal, participants were reimbursed £30 for their time plus£7.20 additional bonus payment (i.e., chance level performance in thejudgement task, see below).

The fMRI also included a between-subject incentive manipulation, andparticipants were assigned to the experimental conditions in an interleavedmanner. Using the same wording framework as in the behavioural replicationstudy, participants in the incentive group were instructed that they couldreceive an additional 50% on top of their payment for the whole datacollection if they estimated correctly, translating to an additional£0.80 per correct estimate^4^. The study protocol wasapproved by the University of Reading Research Ethics Committee (UREC;18/18).

#### 2.3.2 Material

In the fMRI study, the same magic tricks were presented as before, but thevideo files themselves underwent further editing to optimise them for usagewithin the MRI scanner. Luminance, for instance, was adapted wherenecessary. Furthermore, a mock video was created and added individually tothe beginning of each magic trick. Over a period of 6 s, the first frame ofeach magic trick was displayed, overlaid with a black video that included aviewing focus that gradually opened up to match the viewing focus of themagic trick file. The resulting magic trick files were on average 38.5 slong (SD = 8.63, min = 26.6 s, max = 58.64). The sameframes as described above were used to create cue images.

#### 2.3.3 Tasks & measurements

Overall, the tasks were not substantially changed and only adapted for thefMRI environment. The study protocol included more tasks (seeMeliss et al., 2022); however, hereonly the tasks used for the analyses are described.

##### 2.3.3.1 Magic trick watching task

Participants were asked to perform the magic trick watching task insidethe MRI scanner (seeFig. S1illustrating the trial structure used in the fMRIexperiment). The experiment was displayed on a black background and alltext was presented in white unless indicated differently. The beginningof the display of each magic trick video was synced with the scanner TTL(transistor-transistor logic) pulse at the beginning of each repetitiontime (TR). A jittered fixation (4-10 s, TTL aligned, only even integers)was displayed in between the end of the magic trick and the estimaterating. Different from the behavioural studies, the percentage sign wasomitted in the answer options and the answer options were displayed incolours matching the button colours on the four-button MRI-compatibleresponse device (https://www.curdes.com/mainforp/responsedevices/buttonboxes/hhsc-1x4-cr.html).Estimate ratings were recorded by pressing the button in the colour ofthe corresponding estimate. There was a fixed response window of 6 s. Ifparticipants chose an estimate sooner, the answer options would turnwhite. After a brief fixation (0.05 s), the curiosity rating wasdisplayed and a random number was highlighted in red. Participants wereinstructed to move the highlighted number to the left or right (usingindex and middle finger, respectively) before confirming their selectionusing the red button. The fixed response window was 5.95 s.

Participants completed two practice trials outside the MRI scanner.Inside the MRI scanner, participants completed 36 trials of the magictrick watching task distributed over three blocks. The order in whichmagic tricks were displayed was pseudo-randomised to control for trialorder effects. Trial orders were simulated so that high and lowcuriosity magic tricks were equally distributed across blocks (low andhigh curiosity magic tricks were defined based on data byOzono and colleagues (2021)) whileno more than four magic tricks of each category could followconsecutively. Furthermore, trial orders were restricted so that themaximum range of Spearman-rank correlations between any two trial ordersdid not exceed a threshold of 0.7. In total, 25 trial orders weresimulated and used once in each group.

Self-paced breaks were offered between each block. Participants wereexposed to the incentive manipulation in written form before the startof the first task block and had to confirm it by pressing a button onthe button box. The incentive manipulation was also repeated verbally bythe experimenters. Before the start of the second and third block, theincentive manipulation was repeated.

##### 2.3.3.2 Surprise recall and recognition task

No changes were made with respect to the memory task.

##### 2.3.3.3 Task motivation inventory (TMI)

The TMI was completed inside the MRI scanner at the end of theexperiment. Items were displayed in random order, andparticipants’ responses were collected akin to the curiosityratings.

#### 2.3.4 Experimental procedure

After screening procedures and pre-scanning assessments (described elsewhere,Meliss et al., 2022),participants were invited to an fMRI scanning session at the Centre forIntegrative Neuroscience and Neurodynamics (CINN) at the University ofReading for a 2-h session. Practice and experiment were presented usingPsychophysicsToolbox (PTB) 3 (Brainard,1997) with GStreamer media framework run on Matlab on a 13-inchApple MacBook. Practice trials were completed outside the MRI scannerlooking directly at the screen, whereas back projection was used during theexperiment. Before and after the magic trick watching task, resting-statedata (10 min, eyes open) were acquired. At the end of the experiment, theTMI was presented during which the anatomical sequence was run. Thefollow-up memory test was conducted online: One week later, participantsreceived the link to the surprise memory assessment executed usingCollector.

### 2.4 Data pre-processing and analysis

#### 2.4.1 Behavioural data

Behavioural data from each data collection were processed and analysed in thesame way. All behavioural pre-processing and analysis were carried out in R3.6.3 (R Core Team, 2020).

To test for between-group differences in motivation (TMI scores as well asratings of curiosity obtained in the magic trick watching task), data fromthe TMI were analysed using Welch’s Two-Sample t-tests. Curiosityratings for the magic trick movies were analysed using Linear Mixed Effects(LME) models with the*lme4*package (Bates et al., 2015) specifying a fixed effect forincentives (effect-coded; -1 = control group, 1 = incentivegroup) and random effects for intercepts of participants and stimuli.

Data from the recognition block were dummy-coded by comparing the chosenresponse to the correct answer. Additionally, recognition performance wascombined with confidence ratings. Specifically, a correct answer chosen witha confidence larger than three was coded as correct for “highconfidence recognition”, which should partly reflect arecollection-based recognition memory measurement (Yonelinas, 2001,^2002^). For the recall performance of the answers collected inthe cued recall paradigm, a script was used to assign 0 to all answersmatching “no recall” (or variants thereof). All remaininganswers were coded by the same rater across all three data collections. Amagic trick was rated as recalled if the change that occurred during thetrick was accurately remembered, and the coder had a several meetings withone of the authors to discuss the coding criteria. To examine thereliability of the coding, another independent rater coded 10% (randomlyselected) of the recall descriptions from the first behavioural study. Theinter-ratter reliability was found to be low-moderate (kappa = 0.47).However, it is important to note that our main analyses were focused on therecognition memory task.

Our main analyses focused on the effects of curiosity, monetary incentives,and their interaction on memory encoding. Encoding data were analysed usinga meta-analytic approach. For each data collection, Generalised LME (gLME)models were applied specifying fixed effects for curiosity, incentives, andtheir interaction as well as random effects for the participant and stimulusintercept and random slopes for the curiosity effect. Curiosity ratings weremean-centred within each participant and incentive manipulation was againeffect-coded. The same model was run on three different memory thresholds:correct recognition (regardless of confidence), high confidence recognition,and cued recall. To further investigate whether incentives and curiosityinfluence the quality of memory in an exploratory analysis, wesystematically varied the confidence cut-off, creating additional dependentvariables (recognition with confidence >0 through to recognition withconfidence >5) and applied the same gLME model as described above.The unstandardised parameter estimates from the gLME models (i.e., betaestimates and standard errors) from each data collection were extracted andsubmitted to a fixed-effect meta-analysis (weighted least squares) using the*metafor*package (Viechtbauer, 2010) to integrate individual coefficients from thethree data collections.

#### 2.4.2 fMRI data

##### 2.4.2.1 fMRI acquisition and pre-processing

fMRI data were obtained in a 3.0 T Siemens Magnetom Prisma scanner with a32-channel head coil. Whole-brain images were acquired (37 axial slices,3 x 3 x 3 mm, interslice gap of 0.75 mm) using an echo-planarT2*-weighted sequence (TR = 2000 ms, echo time = 30ms, field of view: 1344 x 1344 mm^2^, flip angle:90°).

Pre-processing steps included B_0_distortion correction,despiking, slice-timing and head motion correction, and normalisation toMNI space using the ICBM 2009c Nonlinear Asymmetric Template.Additionally, data were smoothed to achieve an approximate smoothness offull width half maximum kernel of 8 mm and time series were scaled to amean of 100. Local white matter time series, the first three principalcomponents of the lateral ventricles, as well as motion estimates, wereincluded as regressors of no interest to denoise the data. During linearregression, time courses were also band-pass filtered for frequenciesbetween 0.01 and 0.1 Hz. Time points were censored (i.e., set to zero)if the Euclidean norm of per-slice motion exceeded 0.3 mm or if morethan 10% of brain voxels were outliers.

##### 2.4.2.2 Intersubject correlation (ISC) analysis

Due to increased stimulus complexity in naturalistic paradigms, theapplicability of traditional analysis methods developed for task-basedfMRI relying on specifying onset and duration of stimuli (e.g., generallinear models; GLMs) is limited and model-free approaches are usedfrequently (Sonkusare et al.,2019). One of these data-driven methods is intersubjectcorrelation (ISC;Hasson et al.,2004). Here, the assumption is that the brain response whenperceiving and processing naturalistic stimuli is composed of astimulus-driven signal as well as spontaneous activity unrelated to thestimulus (Nummenmaa et al.,2018). The stimulus-driven signal is time-locked to the stimuliand shared across subjects whereas the intrinsic fluctuations arecancelled out as noise. To determine brain areas that encode informationabout the presented stimuli consistently across subjects, the timecourse of a given voxel in subject A is correlated with the time courseof the same voxel in subject B. This is repeated for each voxel in thebrain for each pair of participants in the sample, creating pairwise ISCmaps.

During the magic trick watching task, the beginning of each magic trickvideo was aligned with the beginning of a TR. Likewise, the jitteredfixation after the magic trick presentation was aligned with thebeginning of a TR and presentation times and response windows weremultiple of the TR. These steps were undertaken to allow that the timeseries could be concatenated (seeFig.2A) to (a) remove volumes of no interest, (b) reorder thevolumes so that the concatenated order would remain invariant acrosssubjects irrespective of the pseudo-randomised order in which the magictricks were presented (seeThomas etal., 2018), and (c) account for the delay in the hemodynamicresponse function (HRF) by shifting the time course. Volumes acquiredduring the mock video presentation, fixation and estimate/curiosityratings were considered as volumes of no interest because ISC criticallyrelies on subjects receiving the same time-locked stimuli and transient,non-specific activity can be found at stimulus onset (Nastase et al., 2019).

**Fig. 2.**
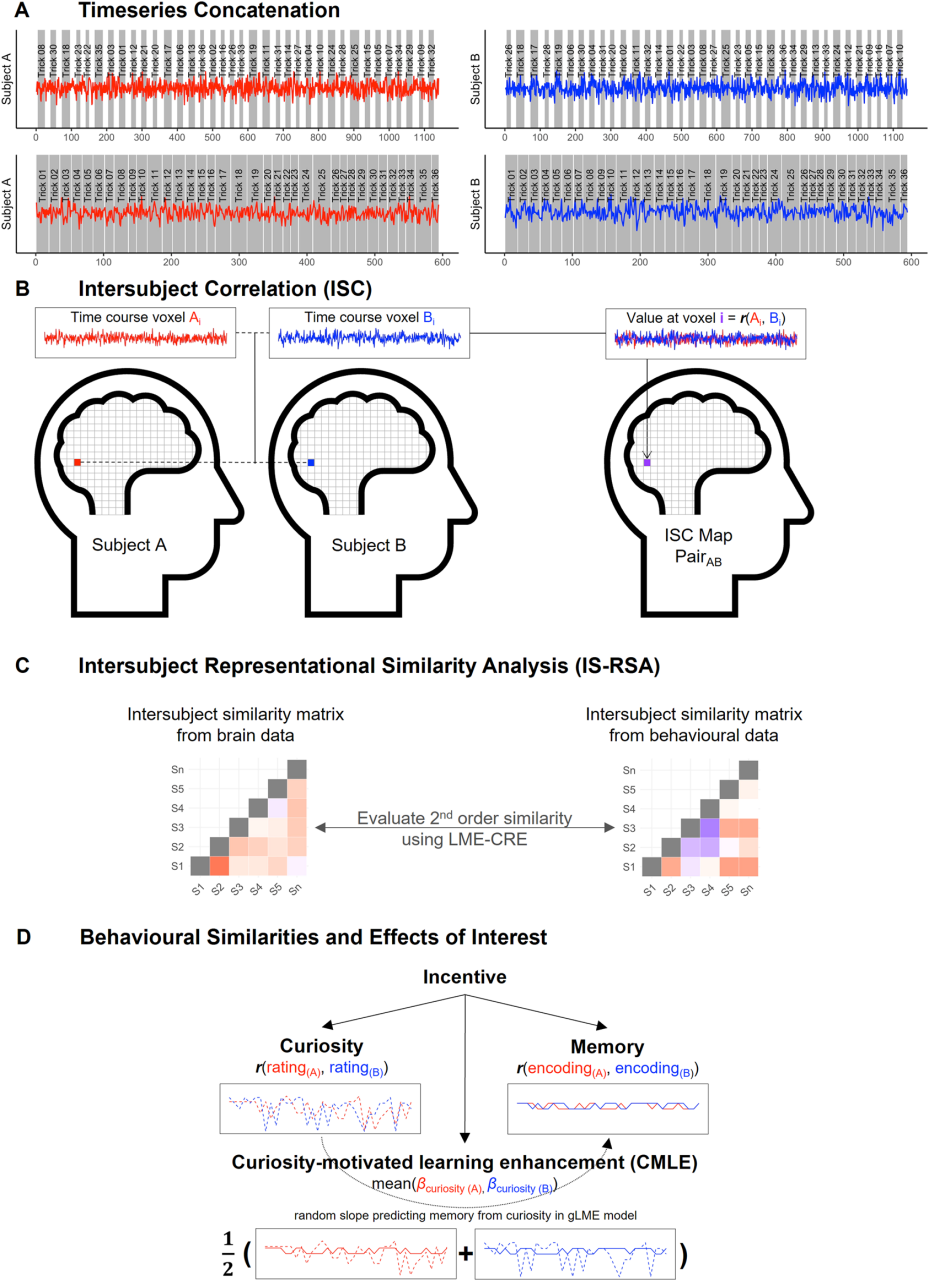
Illustration of processing and analysis methodology within theintersubject framework.*Note.*To account forthe dynamic nature of the stimuli, intersubject correlation(ISC) analysis was applied. (A) In the first step, data wereconcatenated to remove volumes of no interest, reorder volumes,and account for the lag in the HRF. (B) The concatenated timeseries of each voxel were correlated for each pair ofparticipants creating pairwise ISC maps representing similarityin the brain response between participants (figure adapted fromNastase et al.,2019). (C) To anchor idiosyncratic response patterns tobehavioural measurements, intersubject representationalsimilarity analysis (IS-RSA) was used to relate similarities inthe brain response to similarities in behavioural measurements(figure adapted fromFinn etal., 2020). (D) Behavioural measures of interest werecuriosity, encoding, and curiosity-motivated learningenhancement (CMLE). To determine behavioural similarities incuriosity and encoding, the time course of rating and encodingwere correlated for each pair of participants. For CMLE, eachsubject’s random slope predicting memory encoding withcuriosity estimated by the behavioural gLME was extracted andthe mean as a non-parametric difference measure was calculatedfor each pair.

As assumptions regarding the duration of the HRF lag to account for inISC analyses vary (Hasson et al.,2004;Nummenmaa et al.,2012;Zadbood et al.,2017), a preliminary intersubject pattern correlation (ISPC;J.Chen et al., 2017)—aspatial form of ISC—was computed to determine the optimal HRFlag. This preliminary analysis indicated the optimal HRF lag to be 4 TRs(seeSupplementaryMaterial and Fig. S2). We also examined the consistency ofISC across different lags, with the results showing that ISC was notstrongly affected by varying lags (seeSupplementary Materialand Fig. S3), suggesting that our results are robustirrespective of how lags are determined. The concatenated time seriesconsisting of 594 volumes were correlated for each pair of participants(using AFNI’s“*3dTcorrelate*”*,*Fig. 2B). Each correlation thus hada (non-independent) sample size of 594 volumes. This procedure resultedin 1225 pairwise ISC maps, which were then subjected to Fisher’s*z*-transformation for further analysis.

To determine brain areas showing significant synchronicity betweensubjects, linear mixed-effect models with crossed random effects(LME-CRE;G. Chen et al., 2017)were specified to predict the pairwise Fisher’s*z*-transformed ISC maps (using AFNI’s“*3dISC”*). The LME-CRE framework doesnot only account for the interrelatedness in the pairwise ISC map databy specifying crossed random intercepts for both subjects in each pairbut also offers analytical flexibility to specify grouping variables toinvestigate the effects of incentives on ISC during magic trick watchingas well as of other covariates (see below). To specify the fixed effectof incentives, deviation coding was adopted where 0.5 was assigned tosubjects in the control group and -0.5 was assigned to subjects in theincentive group. By adding up these values for each pair, group wasdefined as 1 (both subjects in control group), 0 (both subjects indifferent groups), or -1 (both subjects in the incentive group).

##### 2.4.2.3 Intersubject representational similarity analysis (IS-RSA)

Nastase and colleagues (2019)proposed a formal definition of ISC, where they divided thestimulus-driven component further into processes consistent acrosssubjects and idiosyncratic responses, that are nonetheless induced bythe stimulus but characterised by timings and intensities specific toeach subject. The consistent response can be estimated by averaging theISC, given that subject-specific and spontaneous responses will averageout. To quantify the subject-specific responses in the time courses,other known information about the subjects can be used to“anchor” the response—an approach known asintersubject representational similarity analysis (IS-RSA;Finn et al., 2020;Nummenmaa et al., 2012). More specifically,the similarity in participants’ behavioural data (e.g., traitscores,Finn et al., 2018; age,Moraczewski et al., 2018;recall performance,Nguyen et al.,2019; behavioural ratings,Nummenmaa et al., 2012) can be used to predict thesimilarity in the brain response (Fig.2C) by, firstly, calculating subject-by-subject similaritymatrices separately for behavioural and brain data. In a second step,the geometry of both matrices can be compared or matched correlationallybased on the second-order isomorphism within representational similarityanalysis (RSA;Kriegeskorte et al.,2008). The second-order similarity can be evaluated usingLME-CRE. Importantly, the pseudo-randomisation of trials allows forsimilarities in brain responses between participants to be attributed tothe behavioural anchor, rather than to similarities in the trialorder.

Here, we were interested in how similarity in (1) curiosity, (2) memoryencoding, and (c) curiosity-motivated learning enhancement (CMLE)predicts similarity in the neural responses across subjects (Fig. 2D). To calculate thesubject-by-subject similarity matrices in the first two instances, thetrial-by-trial values (subject-wise mean-centred curiosity ratings anddummy-coded encoding performance on the high confidence criteria,respectively) were correlated for each pair of participants (afterre-ordering the values for each subject to account for thepseudo-randomisation). To control for potentially shared variancebetween the similarity matrix of curiosity and the similarity matrix ofmemory, Fisher’s*z*-transformed pairwisecuriosity correlations were residualised by removing the proportion ofvariance that can be linearly predicted by Fisher’s*z*-transformed pairwise memory correlations.Likewise, Fisher’s*z*-transformed pairwise memorycorrelations were residualised by removing the proportion of variancethat can be linearly predicted by Fisher’s*z*-transformed pairwise curiosity correlations. In doingso, the unique effects of curiosity and memory could beinvestigated.

CMLE was quantified by extracting the individual curiosity beta values(estimated by the specification of random slopes predicting memory withcuriosity) from the gLME model for high confidence recognition andmean-centring them^5^. The beta valuequantifies the magnitude of the association between curiosity and memoryfor each individual. As there was only one value per subject (ratherthan a time course), the similarity matrix was calculated using the AnnaKarenina (AnnaK) model, providing a metric reflecting the absoluteposition on the scale, that is, the mean of both subjects (Finn et al., 2020). This ispreferable compared to using a relative distance metric like theEuclidean distance, as it allows for a scenario where low scoringsubjects are more similar to other low scoring ones, but high scoringsubjects are less similar to each other. Previous studies using workingmemory in IS-RSA found that the AnnaK model fitted the data better thanthe Euclidean distance and yielded to higher replicability betweensamples (Finn et al., 2020).Another benefit of using the mean is that effects in both directions canbe captured: if the correlation between brain and behaviour is positive,then high scorers are alike and low scorers different, whereas anegative sign indicates that low scorers are alike and high scorersdifferent.

To link idiosyncratic responses in the stimulus-driven brain response tothe behavioural effects of interest, LME-CRE were used to predict thepairwise Fisher’s*z*-transformed ISC maps. Asdescribed above, separate models were estimated for unique curiosity,unique memory, and CMLE, again specifying crossed random intercepts forboth subjects in each pair. Fixed effects were specified for group(incentive vs. no incentive, effect-coded), the respective behaviouralsimilarity (of curiosity, memory, or CMLE) as well as their interaction.Behavioural similarity was grand-mean centred before computing theinteraction term with the group variable.

##### 2.4.2.4 Thresholding and regions-of-interest (ROI) approach

All analyses were conducted at the whole-brain level specifying a greymatter (GM) mask: during pre-processing, each subject’s greymatter (GM) mask based on FreeSurfer parcellation was transformed toecho-planar imaging (EPI) resolution. After averaging the masks acrossthe sample, the mean image was thresholded so that a voxel was includedin the group GM mask if it was GM in at least 50% of the sample. Thisthreshold was chosen to ensure that our analysis focused on brainregions consistently identified as GM across the majority of the sample,thereby enhancing the reliability of our findings. By applying thisaveraged GM mask, only 3% of the total voxels were excluded from the keybrain regions (i.e., ROI masks described below), a minimal reductionthat is unlikely to significantly impact our overall analysis.

To account for the multiple testing problem due to mass-univariatetesting, cluster-extent based thresholding was performed using therecommended initial threshold of*p*value = 0.001(Woo et al., 2014). Theresulting map was cluster-extent corrected based on the output ofsimulations performed using “3dClustSim” for first nearestneighbours clustering (NN = 1; faces of voxels touch) and acluster threshold of*α*= 0.05 resultingin a threshold of*k*= 20 voxels. This thresholdis aimed to balance the sensitivity and control for false positiveresults, while we should bear in mind that smaller brain areas might notbe detectable. Unthresholded statistical maps were uploaded toNeuroVault (https://neurovault.org/collections/12980/).

In addition to whole-brain analysis, we were also interested in regionspreviously implicated in motivated learning and*aprior*i defined the following regions-of-interest (ROIs): aHPC,NAcc, CN, and VTA/SN. The aHPC has been chosen as increased activity forremembered compared to forgotten items is predominantly centred inanterior parts of the HPC (Kim,2011;Spaniol et al.,2009). The aHPC is also sensitive to the effects ofincentives and motivationally relevant information on encoding (Adcock et al., 2006;Poppenk et al., 2013). To create the aHPCROI, AFNI’s “whereami” was used to extract thebilateral HPC from the Glasser Human Connectome Project atlas (Glasser et al., 2016). Followingthe recommendations byPoppenk et al.(2013), the aHPC was created by using the MNI coordinate y= 21P to determine the uncal apex as a landmark to divideanterior and posterior HPC (“3dZeropad”). To create ROImasks for NAcc, CN, and VTA/SN, atlaskit (https://github.com/jmtyszka/atlaskit) was used to extract theNAcc, CN, Substantia Nigra pars reticulata (SNr), Substantia Nigra parscompacta (SNc), and Ventral Tegmental Area (VTA) from a high-resolutionprobabilistic subcortical nuclei atlas in MNI space (Pauli et al., 2018) specifying a probabilitythreshold of 15%. This is similar to procedures by others presentingmagic tricks inside the MRI scanner (Lauet al., 2020). To create the VTA/SN mask, the masks for VTA,SNr, and SNc were combined. In total, the aHPC mask contained 162voxels, the CN mask contained 573 voxels, and the NAcc and VTA/SN maskboth contained 60 voxels each (seeFig. S4). Tocorrect for multiple comparisons within each ROI, False Discovery Rate(FDR) correction was applied at*q*= 0.05.Additionally, clusters were thresholded at*k*= 5(NN = 1). ROI masks can be accessed in the NeuroVault collection(https://neurovault.org/collections/12980/).

## 3 Results

### 3.1 Behavioural data

The groups did not differ in their motivation in any TMI scale in any of theassessments (all*p*> 0.09). Likewise, no groupdifference was observed in the curiosity ratings (all*p*> 0.199). The detailed results for TMI scores and curiosity ratings canbe found inTable S1 andS2in the Supplementary Material, respectively.

Next, we investigated the effects of curiosity, incentives, and their interactionon memory encoding specifying the same gLME model for each data collection andfor each type of memory measurement (namely, recognition, high confidencerecognition, and cued recall). We then submitted the parameter estimatesobtained from these models to fixed effects meta-analyses, conducted separatelyfor each memory measurement. The results of the fixed effects meta-analyses areshown inTable 2, with results from eachdata collection presented inTable S3. Curiosity had a positive effect on memory encoding: magictricks for which participants reported higher curiosity were more likely to beencoded. While the overall curiosity effect was not significant for recognitionper se, significant effects were observed for high confidence recognition andcued recall. With respect to the effect of monetary incentives on memoryencoding, the effects were overall positive, that is, participants in theincentive group were more likely to encode the magic tricks compared toparticipants in the control group. However, the overall effect only reachedsignificance for the high confidence recognition memory measurement. Theinteraction between monetary incentives and curiosity did not reach significancefor any of the memory thresholds investigated.

**Table 2.** Integrated results of gLME models predicting memory encoding usingcuriosity, monetary incentive, and their interaction.

	*b* ( *SE* )	OR [95%-CI]	*z* value	*p* value
Curiosity				
Recognition	0.023 (0.023)	1.02 [0.98; 1.07]	0.988	0.323
High confidence recognition	0.084 (0.022)	1.09 [1.04; 1.14]	3.766	< 0.001
Cued recall	0.098 (0.025)	1.10 [1.05; 1.16]	3.842	< 0.001
Monetary incentive				
Recognition	0.084 (0.050)	1.09 [0.99; 1.20]	1.676	0.094
High confidence recognition	0.155 (0.067)	1.17 [1.03; 1.33]	2.336	0.019
Cued recall	0.119 (0.075)	1.13 [0.97; 1.30]	1.599	0.110
Interaction				
Recognition	-0.002 (0.022)	1.00 [0.96; 1.04]	-0.070	0.944
High confidence recognition	-0.010 (0.021)	0.99 [0.95; 1.03]	-0.479	0.632
Cued recall	-0.025 (0.024)	0.98 [0.93; 1.02]	-1.058	0.290

*Note*. Separate models were run for each memorythreshold. gLME = Generalised Linear Mixed Effects. SE= standard error. OR = Odds Ratio, CI =confidence interval.

Subsequently, we examined the quality of recognition memory by changing theconfidence cut-off threshold gradually (0 ≤ cut-off ≤ 5). Again,the same gLME model was run for each confidence threshold and each datacollection and estimates were integrated using a fixed-effects meta-analysis(for detailed results for each effect on each threshold, seeTable S4) to extractthe integrated b estimates for each effect at each confidence cut-off. Then, toexamine how the cut-off is related to memory enhancement effect, the integratedfixed effects b estimates were predicted by the confidence cut-off in a linearmodel separately for each effect. The cut-off was scaled from 0 to 5 so that theintercept is interpretable.

The results of the exploratory analysis are illustrated inFigure 3, and the detailed regression table can be found inTable S6. Morespecifically, they show that when calculating a linear regression to predict theintegrated curiosity effect b values based on the confidence cut-off, theconfidence cut-off was a significant predictor in the model (*B*= 0.021, 95%-CI [0.011; 0.031],*p*= .004)indicating that the integrated curiosity effect increases as the confidencecut-off increases: the Odds Ratio (OR) of the curiosity effect was 1.02 forconfidence cut-off = 0 and 1.12 for confidence cut-off = 5.

**Fig. 3.**
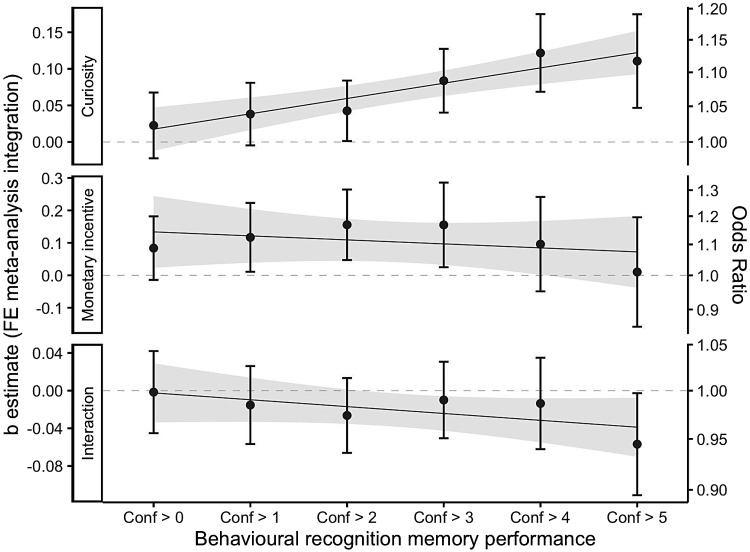
Integrated fixed effects of curiosity, monetary incentive, and theirinteraction as a function of confidence cut-off.*Note.*The x-axis shows the gradual confidence cut-off, and y-axis illustratesthe integrated effect size (left—unstandardised,right—OR). Each panel shows one of the fixed effects specified inthe gLME model. The integrated b estimate for each effect and confidencethreshold is plotted and error bars indicate 95%-CI. The regression lineillustrates the linear model predicting the effect with the gradualconfidence cut-off.

However, in the model predicting the integrated monetary incentive effect bvalues with the confidence cut-off, the cut-off was not a significant predictorin the model (*B*= -0.012, 95%-CI [-0.049; 0.024],*p*= .402). Likewise, using a linear model to predictthe integrated interaction effect b values using the confidence cut-off,confidence cut-off was not a significant predictor (*B*=-0.007, 95%-CI [-0.018; 0.003],*p*= .122).

The results suggest that only the curiosity effect, but not the monetaryincentive or the interaction effect, is sensitive to the confidence cut-off.More specifically, they show that the more confidently participants recognisethe correct answer option, the larger the effect of curiosity on encoding.Monetary incentive and interaction effect, on the other hand, remain invariantregarding the confidence thresholds.

The results of all 21 individual gLME models (seven memory measurements in threeexperiments) can be found inTable S4. Additionally,Figure S5contains the equivalent ofFigure 3plotting the effects from each data collectionindividually. Because 8 of 21 gLME models produced a singular fit warning duringexecution, all analyses were repeated using a simplified gLME model with areduced random effects structure omitting the random slopes for the curiosityeffect. Applying this reduced gLME model, however, did not affect the results ofthe meta-analyses and associated confidence cut-off linear model (seeTable S5, S6, and S7aswell asFig. S6 andS7).

### 3.2 fMRI data

#### 3.2.1 Intersubject correlation (ISC)

ISC analyses were carried out to identify brain areas with activity driven bymagic trick watching. Significant ISC was found bilaterally in all four ROIs(aHPC, VTA/SN, NAcc, and CN; seeFig. S8). Shared activity measured as significant ISCin the reward network has previously been observed in naturalistic viewingparadigms when presenting comedy movie clips to participants (Jääskeläinen et al.,2016).

**Fig. 4.**
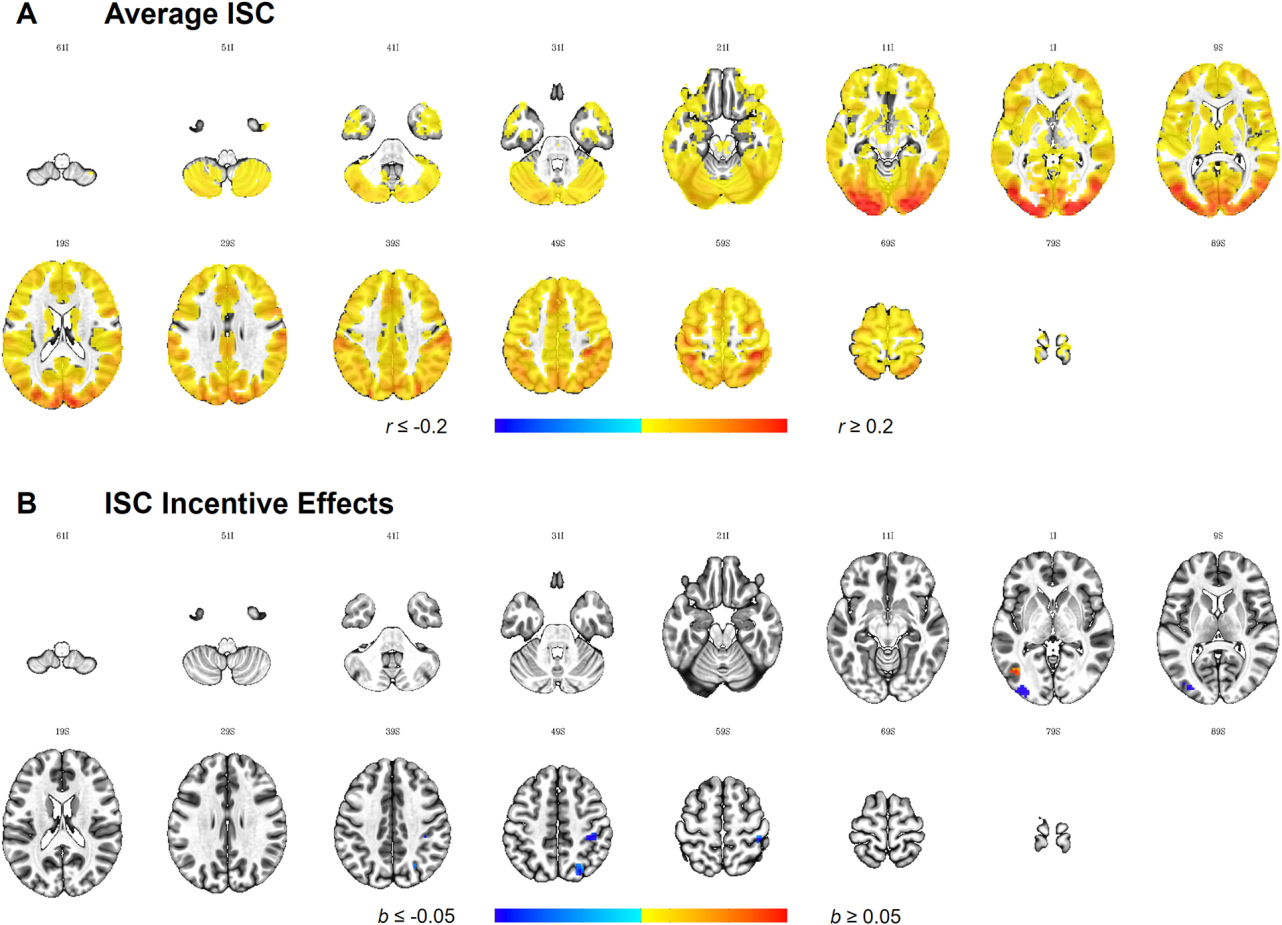
Whole brain ISC and incentive effects therein.*Note*.Results are thresholded at*p*< 0.001,cluster-extent corrected at*k*= 20(equivalent to per-cluster α = 0.05), and plotted onthe ICBM 2009c Nonlinear Asymmetric Template. Images are displayedin neurological orientation, where the left side of the brain isdepicted on the left side of the image. The first two digits at thetop of each image represent the*z*axis value forMNI coordinate, followed by the sign of the number, I = -(inferior), S = + (superior). While (A) highlightswidespread ISC across cortical and subcortical areas during magictrick watching across both groups, (B) shows clusters where the ISCis higher in the incentive group compared to the control group inblue, and a cluster where ISC is higher in the control group inred.

At the whole-brain level, widespread cortical and subcortical synchronisation(Fig. 4A,Table S8) wasobserved, especially dominant in the bilateral visual cortex as well asbilateral parietal somatosensory (BA 2, BA 5, BA 40, BA 1/2/3) and inattention-related areas (BA 7 and BA 39) as well as bilateral premotor andsupplementary motor areas (BA 6, BA 8). Overall, this is in line with otherstudies showing that dynamic stimuli synchronise brain activity in visualareas (e.g.,Aliko et al., 2020;Baldassano et al., 2017;Hasson et al., 2004;Nguyen et al., 2019), but also with prepositionslinking motor and somatosensory areas to the observation of actions (Keysers et al., 2010;Thomas et al., 2018). Likewise, the decline ofthe ISC from posterior to anterior as well as from lateral to medial areasin the brain can be attributed to higher intersubject variability in thestimulus-induced response in “intrinsic systems” (e.g.,prefrontal and cingulate cortices;Ren etal., 2017).

We also investigated whether the availability of incentives had an effect onthe ISC. While no effects were found in the ROIs, four clusters were foundat the whole-brain level (Fig. 4B,Table S8).More specifically, in the incentive group, we found higher ISC in areas inthe left middle occipital gyrus, right postcentral gyrus (BA 2), and rightintraparietal sulcus (IPS). Higher ISC in the control group was observed inthe left lateral occipital cortex (Area V5/MT+).

#### 3.2.2 Intersubject representational similarity analysis (IS-RSA)

IS-RSA were carried out to identify brain regions with intersubject temporaldynamics, reflecting the intersubject variability in our behavioural effectsof interest as well as brain regions where this association was influencedby the incentive manipulation. For this purpose, for each behavioural effectof interest, an LME-CRE model was specified with fixed effects for group,behavioural similarity, as well as their interaction. The inclusion of thecovariate and the interaction effect did not affect the main effect ofincentive (all correlations with unthresholded incentive effects reportedabove ≥ 0.92), hence the incentive effects are not further discussed.Below, the main effects of each behavioural variable are described beforediscussing the interaction effects and results for the ROI analysis arereported followed by whole brain analysis.

##### 3.2.2.1 IS-RSA for each behavioural effect of interest

Here, the main effects of each behavioural variable are reportedhighlighting clusters where the behavioural similarity matrix waspredictive of the neural similarity matrix. The underlying assumption isthat participants similar in behavioural effects of interest (e.g.,curiosity ratings) will process the magic trick videos more similarlyand regions involved in these processes will reflect this similaritycorrespondingly and hence are detected in this analysis.

###### 3.2.2.1.1 Curiosity effect

**Fig. 5.**
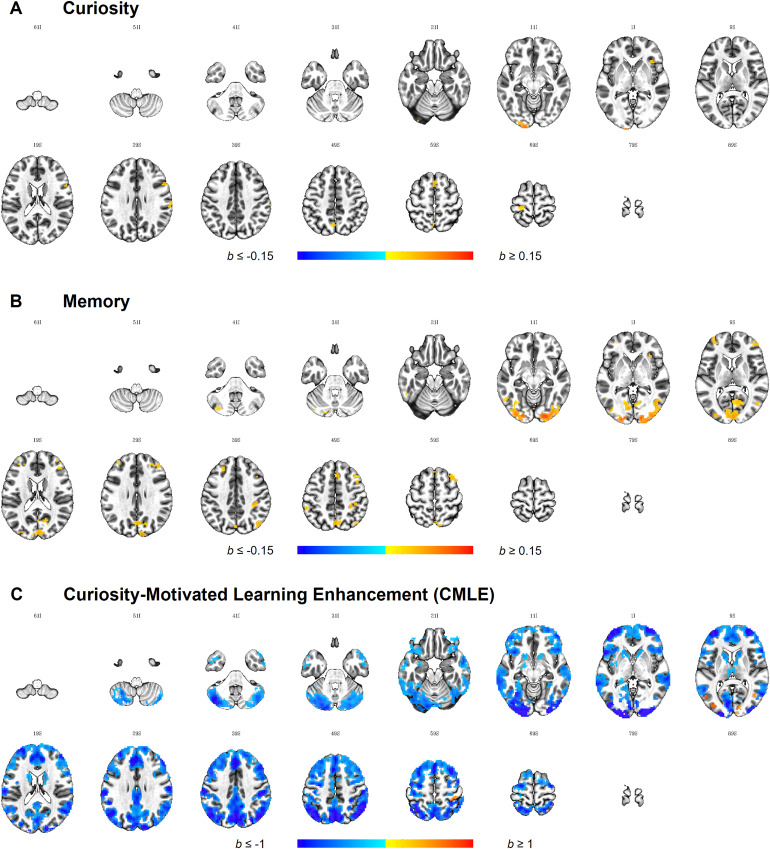
Whole-brain IS-RSA for each behavioural effect of interest.*Note*. Results are thresholded at*p*< 0.001, cluster-extentcorrected at*k*= 20 (equivalent toper-cluster α = 0.05), and plotted on the ICBM2009c Nonlinear Asymmetric Template. Images are displayed inneurological orientation, where the left side of the brainis depicted on the left side of the image. The first twodigits at the top of each image represent the*z*axis value for MNI coordinate,followed by the sign of the number, I = - (inferior),S = + (superior).

The curiosity effect was defined as the pairwise correlation oftrial-by-trial curiosity ratings. Importantly, we here used uniqueeffects of curiosity where Fisher’s*z*-transformed pairwise curiosity correlations wereresidualised by removing the proportion that can be linearlypredicted by Fisher’s*z*-transformed pairwisememory correlations. No activity in the four ROIs survivedthresholding. At the whole-brain level, seven positive clusters werefound (Fig. 5A,Table S9)where idiosyncratic patterns in curiosity were anchored to the brainresponse. These clusters were located in the left primary visualcortex (V1), right inferior frontal gyrus (pars opercularis),bilateral supplementary motor area (BA 8), left postcentral gyrus(primary somatosensory cortex), left precuneus (BA 7), rightanterior insula cortex (AIC), and right supramarginal gyrus (BA40).

###### 3.2.2.1.2 Memory effect

**Fig. 6.**
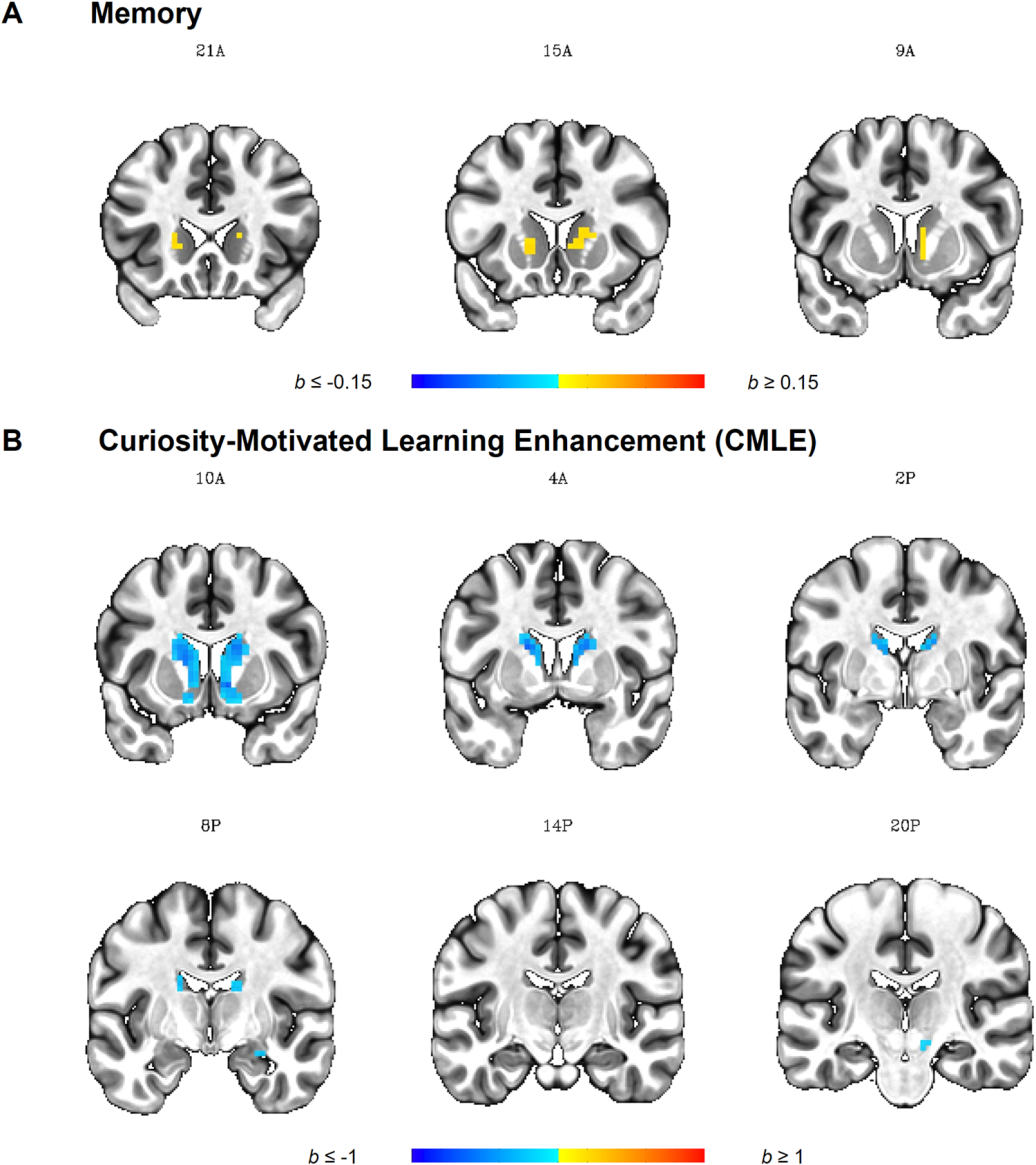
Effects of memory and CMLE in the ROIs.*Note.*Results are FDR-corrected at*q*< 0.05, cluster-extentcorrected at*k*= 5, and plotted onthe ICBM 2009c Nonlinear Asymmetric Template. Images aredisplayed in neurological orientation, where the left sideof the brain is depicted on the left side of the image. Thefirst two digits at the top of each image represent the*y*axis value for MNI coordinate,followed by the sign of the number, P = -(posterior), A = + (anterior).

The memory effect was defined as pairwise correlation oftrial-by-trial encoding performance ratings. Again, the uniquecontribution of memory was investigated akin to what was describedabove in the context of curiosity. Similarity in brain responsecould be anchored to similarity in memory encoding in a bilateralcluster in the CN ROI (Fig. 6,TableS9); however, no effects were observed for the otherthree ROIs. At the whole-brain level, 21 clusters were found (Fig. 5B,Table S9).More specifically, similarity in memory encoding positivelypredicted similarly in brain response bilateral visual areas as wellas the left cerebellum, the bilateral superior (BA 46, BA 9-46,medial BA 8) and middle frontal gyrus (BA 6, BA 8), precuneus (BA7), and lateral parietal areas including the right angular gyrus (BA39) and somatosensory areas (BA 2, BA 40), the left lateral temporalgyrus (BA 37, fusiform and inferior temporal gyrus), right middleoccipital gyrus (Area V5/MT+), and the right AIC.

###### 3.2.2.1.3 Curiosity-motivated learning enhancement (CMLE) effect

CMLE was defined based on the random slope predicting memory fromcuriosity in the gLME model, and individual values were extracted.Using the AnnaK model to determine the behavioural similaritymatrix, the prediction was tested whether participants high in CMLEshare similar patterns of brain activity while people low in CMLEshow more variability and vice versa (rather than testing for brainareas where similarity is predicted by similarity in CMLE in alinear fashion).

In the ROI analysis, IS-RSA CMLE were found in all four ROIs (Fig. 6B,Table S9),all of them in a negative direction suggesting that participantswith high CMLE scores had less similar brain activity compared toparticipants with low scores. More specifically, clusters wereidentified in the right aHPC, right VTA/SN, bilateral CN, andbilateral NAcc (Fig. S9for scatter plots). Additionally, 15 clusterssurvived cluster-extent thresholding at the whole-brain level, outof which 5 were positively and 10 negatively directed (Fig. 5C,Table S9).Positive clusters were located in the bilateral middle temporalgyrus, the left middle occipital gyrus, the right calcarine gyrus,and the right postcentral gyrus. In these positive clusters,subjects high in CMLE are more alike than subjects low in CMLE whoare more different in their brain response.

In negative clusters, on the other hand, subjects low in CMLE aremore alike and subjects high in CMLE are more different. Negativeclusters were spread across large portions of the brain, insubcortical (e.g., striatum and thalamus) as well as cortical areasalong the anterior and posterior midline (e.g., ACC, SMA, superiormedial gyrus, precuneus, PCC, and cuneus), visual cortex,cerebellum, postcentral gyrus and posterior parietal cortex (PPC),the bilateral middle temporal gyrus, bilateral anterior insulacortex (AIC), as well as dorsolateral prefrontal cortex (dlPFC;centred around the MFG) and anterior PFC stretching into the frontaloperculum/anterior insula (fO/aI).

##### 3.2.2.2 IS-RSA for the interaction between the incentive manipulation and each behavioural effect of interest

Due to the inclusion of group as a fixed effect in the LME-CRE model, itwas possible to determine brain areas where the behavioural similaritymatrix predicted the neural similarity matrix differently depending onthe availability of monetary incentives. In doing so, clusters could beidentified where the behavioural effect is only predictive in one groupor more strongly predictive in one group.

###### 3.2.2.2.1 Curiosity incentive interaction

**Fig. 7.**
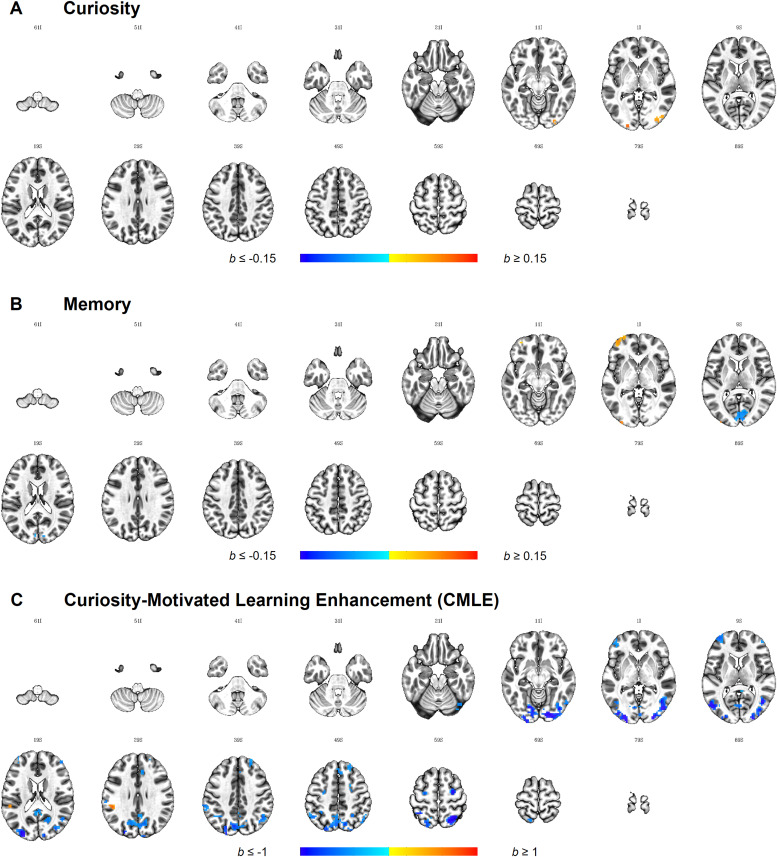
Whole-brain IS-RSA for the interaction between the incentivemanipulation and each behavioural effect of interest.*Note*. Results are thresholded at*p*< 0.001, cluster-extentcorrected at*k*= 20 (equivalent toper-cluster α = 0.05), and plotted on the ICBM2009c Nonlinear Asymmetric Template. Images are displayed inneurological orientation, where the left side of the brainis depicted on the left side of the image. The first twodigits at the top of each image represent the*z*axis value for MNI coordinate,followed by the sign of the number, I = - (inferior),S = + (superior). Positive clusters (shown inred) indicate more positive values in the control comparedto the incentive group, whereas negative clusters (shown inblue) indicate more positive values in the incentivegroup.

When looking at whether the incentive manipulation influences howsimilarity in curiosity predicts similarity in the brain response inthe*a priori*defined ROI, no clusters survivedthresholding. At the whole-brain level, two clusters in thebilateral occipital cortex survived thresholding (Fig. 7A,Table S10).In both clusters, similarity in curiosity was more predictive ofsimilarity in the neural responses during magic trick watching inthe control compared to the incentive group.

###### 3.2.2.2.2 Memory incentive interaction

ROI analysis did not reveal any clusters where incentive influencedhow similarity in memory predicted the similarity in the neuralresponse. In the whole-brain analysis, three clusters were found(Fig. 7B,Table S10)showing a differential predictive effect of similarity in memorydepending on the availability of monetary incentives: One cluster inthe bilateral Calcarine gyrus showed a more positive predictiveeffect of memory in the incentive compared to the control group. Twoclusters were found where the predictive effect of memory was largerin the control compared to the incentive group. Those were locatedin the left dorsolateral prefrontal cortex (dlPFC, BA 10/BA 46) andleft lateral middle occipital gyrus.

###### 3.2.2.2.3 Curiosity-motivated learning enhancement (CMLE) incentive interaction

While effects of curiosity and memory can be understood in a linearmanner, the similarity matrix for CMLE was computed based on anonlinear AnnaK model formulation, further influencing theinterpretation of any effects observed. More specifically, positiveclusters represent brain regions where CMLE high scorers sharesimilar patterns and low scorers show variability, whereas negativeclusters represent regions where CMLE low scorers share similarpatterns and high scorers show variability.

As with the effects of curiosity and memory, the availability ofmonetary incentives did not affect the relationship between thesimilarity in CMLE and brain activity in any of the ROIs. At thewhole-brain level, 20 clusters were found (Fig. 7C,Table S10). One cluster showed positivevalues indicating that values were more positive in the controlcompared to the incentive group. This cluster was located in theleft supramarginal gyrus, where values were negative in theincentive group but weakly positive in the control group.Additionally, 19 clusters showed negative values in which the valuesin the incentive group were more positive compared to the controlgroup. These clusters were predominantly located in posteriorregions, stretching from the occipital poles towards thetemporo-parietal-occipital junction laterally and the cuneusmedially. In the parietal cortex, clusters were found in theprecuneus as well as the superior parietal lobe. Frontally,bilateral clusters in the MFG were found as well as in the rightsuperior frontal gyrus and the superior medial gyrus stretching intothe ACC.

## 4 Discussion

The goal of the present study was to examine the effects of surprised-based curiosityon incidental encoding using dynamic stimuli (i.e., magic tricks). Further, we wereinterested in the combined effects of curiosity and monetary incentives on memoryand neural responses. Behavioural results from three experiments showed thatcuriosity, caused by the induced violation of expectations and surprise, facilitatedincidental encoding independently of the availability of monetary incentives.However, curiosity and monetary incentives did not interact with one another withrespect to behavioural measures of learning. fMRI analysis, accounting for thedynamic nature of the stimuli, revealed that effects of curiosity elicitation,memory encoding, curiosity-motivated learning enhancement (CMLE), as well asmonetary incentive effects, were associated with activity across widespread corticalareas. Additionally, while the effects of memory encoding and CMLE were supported byactivity within the often implicated mesolimbic regions, within the hippocampal-VTAloop, we did not find any indication that the effects of curiosity elicitation andmonetary incentives were supported by shared, stimulus-induced activity in thoseregions.

### 4.1 Effects of curiosity and monetary incentive on memory

In contrast to the previous studies manipulating monetary reward within thetrivia question paradigm (Murayama &Kuhbandner, 2011;Swirsky et al.,2021), we did not find a significant interaction between curiosityand incentive on any of our main measures of interest (recognition, highconfidence recognition, cued recall). These non-significant interaction effectsmay be explained by the differences in the stimuli— while previous workused trivia questions, with curiosity triggered by knowledge gaps, we examinedmemory for magic tricks with a specific focus on surprised-based curiosity.Another possibility is that our incentive manipulation was ratherindirect—monetary incentives were provided not for the memory performanceper se, but for a task unrelated to memory, that is, a separate judgement task.While our design can disentangle the effects of incentives from those ofmotivation (Murayama & Kitagami,2014), it may have made the extrinsic incentives less salient. Futurestudies should further pursue the condition in which curiosity and incentivemanipulation interact, or not, as seen in other instances where this interactionwas not significant (see also Duan et al., 2020;Halamish et al., 2019).

We found an interesting dissociation between the effect of curiosity andincentives on memory. Specifically, the effects of curiosity on encoding wereonly found in high confidence recognition and cued recall, but not inrecognition regardless of confidence. On the other hand, the effect ofincentives on memory (i.e., evident in high confidence recognition and, to alesser extent, in recognition regardless of confidence) did not seem to beinfluenced by confidence levels. Likewise, our exploratory analysis furtherrevealed that, while the effects of curiosity on memory encoding were amplifiedwith increased confidence in the recognition task, this is not the case for theincentive effects.Yonelinas (2002)distinguished recollection and familiarity aspects of memory. According to thistheoretical perspective, high-confidence recognition and recall performance aremore likely to reflect recollection, whereas recognition regardless ofconfidence is more indicative of familiarity processes. Therefore, thesefindings may suggest that curiosity predominantly affects recollection-based,but not familiarity-based, processes. On the other hand, the influence ofmonetary rewards appears to be less selective, impacting memory encodingregardless of the underlying memory process.

These findings were unexpected but after scrutiny of the literature, they weresomewhat consistent with findings previously reported. For example,Gruber and colleagues (2014)reported thatthe curiosity-related recognition advantage in a delayed memory test wasspecific to confidently recognised faces and did not emerge in overallrecognition rates. These results were replicated with short delays (Galli et al., 2018in Exp. 1, but not inExp. 2;Murphy, Dehmelt, et al., 2021),and it has been suggested that curiosity-related memory facilitation is specificto recollection (Gruber et al., 2019;Murayama & Elliot, 2011; cf.Stare et al., 2018for anexception). On the other hand, studies on incentives/rewards and memory havesuggested that rewards may influence both recollection and familiaritycomponents of memory (Bunzeck et al.,2010,^2012^;Patil et al., 2017; cf.Wittmann et al., 2011). Although not specificallyabout memory effects, the findings are also consistent with a meta-analysisshowing that extrinsic rewards/incentives better predicted*quantity*of performance whereas*quality*was better explained by intrinsic motivation, which is a critical source ofcuriosity (Cerasoli et al., 2014).

### 4.2 Neural correlates of curiosity and incentive-motivated learning within reward-related areas and the hippocampus

fMRI research on the effects of curiosity (Gruberet al., 2014) and monetary rewards/incentives (Murty & Adcock, 2014;Wittmann et al., 2005,^2008^) on incidental encoding has repeatedlyimplicated the striatum, VTA/SN, and hippocampus in motivated learning. Althoughwe found that watching magic tricks led to significant synchronisation of brainactivity across subjects in all of these areas, the incentive manipulation didnot lead to differential synchronisation in these*a priori*defined ROI (aHPC, VTA/SN, NAcc, and CN). While some of the effects of interest(i.e., memory and CMLE) were located within the ROIs, others (i.e., curiosity)were not. Importantly, the interaction between any effects of interest andmonetary incentives were only found outside these brain regions.

The biggest difference between this study and previous studies on the effects ofcuriosity and monetary incentives/rewards on encoding lies in the nature ofstimuli used. Compared to the simplistic, static stimuli used by previousstudies (blurred images, trivia questions), magic tricks have added complexitydue to their dynamic nature. Critically, we analysed the fMRI data from dynamicstimuli based on intersubject synchronisation (or intersubject correlation(ISC);Hasson et al., 2004), focusing onthe intrinsic correlation of the voxel-wise time courses across participants todetermine (clusters of) voxels exhibiting a consistent response to thenaturalistic stimuli (Nastase et al.,2019). The obtained ISC maps were further contrasted betweendifferent types of participant pairs in terms of incentive condition, curiosityrating, memory encoding, and CMLE. As such, the current analysis capturesdifferent types of brain dynamics from the classical approach based on theGeneral Linear Model. In fact, while previous studies repeatedly found theinvolvement of reward network brain areas exactly when curiosity was triggered,the current paradigm captures the brain dynamic related to curiosity as awhole—for example, formation of expectation, violation of expectation,experience of curiosity feeling, and post-effect search for potentialexplanation. Therefore, there is good reason to expect that our results would bedifferent from previous work taking the traditional GLM approach.

For instance, the lack of ISC effects of monetary incentives in reward-relatedstructures does not necessarily imply that there is no difference in brainactivation in response to incentives. In fact, it is possible that theactivation in reward-related structures was overall increased in the rewardcompared to the control group, but such an overall increase would not affect thecorrelation. Using the ISC analysis, we instead tested whether the manipulationof incentives increased or reduced the individual differences in the time coursepattern within a voxel (e.g., voxels within the reward-related structures). Inother words, significant differences in ISC are expected when incentives madeparticipants similarly (or differently) attend and comprehend the magic tricks(Hasson, Furman, et al., 2008), andshould manifest in brain areas that are responsible for the synchronisedpsychological functions (e.g., attention, comprehension). As such, we do nothave a strong reason to believe that the reward network responds in anasynchronised fashion. Similar logic should apply to our IS-RSA analysis of theeffects of curiosity and memory performance and the incentive effectstherein.

An interesting observation from the ROI analysis, however, is that an effect ofmemory was found in the bilateral CN, replicating previous studies linkingdeclarative memory to the CN (Blumenfeld et al.,2011;Schott, 2006). Whilemeta-analyses have linked the CN to reward processing (Diekhof et al., 2012;Sescousse et al., 2013), the CN has also beenimplicated in goal-directed action and learning (for a review, seeGrahn et al., 2008), and more specifically,in error learning (Delgado et al., 2005)and reward-motivated learning (Wittmann et al.,2005). However, even in the absence of feedback or reward, enhancedactivity in the CN has also been found when expectations are violated in amovement observation paradigm (Schiffer &Schubotz, 2011), hence linking the CN to perceptual prediction errors(when “what is happening now” differs from the internallygenerated prediction;Zacks et al.,2007). Enhanced CN activity has further been found when participantswatch magic tricks compared to matched control scenes not violating expectations(Danek et al., 2015), suggesting thatmagic tricks, because they violate expectations, trigger perceptual predictionerrors, signalled in the CN. We here found that similarity in encoding magictricks predicts similarity in CN activity. This suggests that the CN is not onlyimportant in signalling perceptual prediction errors, but might also play a rolein updating internal models, or schemata, by supporting the encoding ofincongruent events [see also exploratory intersubject functional connectivityanalysis (ISFC;Simony et al., 2016) tosupport this view, included in Supplementary Material,Figures S10, S11, S12, andS13andTablesS11-14].

Lastly, significant CMLE effects were observed in all four ROIs, but importantly,these effects were negative. Negative clusters indicate that participants whohave low beta values (i.e., participants in which curiosity did not predictmemory performance) showed more similar brain activation time courses inresponse to the magic trick stimuli. Put differently, in negative clusters, theresponse in the low scorers suggests a more exogenous and stimulus-drivenprocess, whereas the response in high scorers is likely more endogenous andindividual-based—participants who have a high curiosity-memoryassociation have more divergent and diverse time courses between individuals.Using the trivia question paradigm,Gruber andcolleagues (2014)were the first to link the effects of curiosity onincidental encoding (i.e., the interaction between curiosity and memory) toactivity in the bilateral NAcc and the right HPC (but not the left). Likewise,activity in the CN and NAcc supports the effects of curiosity on intentionalencoding (Duan et al., 2020). The current study suggests that these brain areasare involved in curiosity-based memory encoding, but likely in a moretime-varying and idiosyncratic manner.

### 4.3 Curiosity- and incentive-motivated learning outside the reward-related areas and the hippocampus

In addition to the results within the*a priori*ROIs (thereward-related areas and the hippocampus), our whole-brain IS-RSA showed broadernetworks of the brain supporting curiosity, memory, and curiosity-motivatedlearning enhancement (CMLE) than previously implicated. We have included anextended discussion of these results in the Supplementary Material, but here wediscuss two notable findings. First, we found that similarity in the curiosityratings predicted similarity in the brain response in the inferior frontal gyrus(IFG), the supplementary motor area (SMA), and the supramarginal gyrus in theIPL. However, initial fMRI research using the trivia question paradigm suggestedthat curiosity—operationalised as the anticipation of rewardinginformation—is supported in dopaminergic regions in the striatum andmidbrain (Gruber et al., 2014;Kang et al., 2009). The elicitation ofcuriosity has more recently been linked to a state of uncertainty, potentiallydue to a violation of expectations (Gruber& Ranganath, 2019;Murayama etal., 2019). Indeed, both the SMA (Cheung et al., 2019;Volz et al.,2005) and the anterior insula (Grinband et al., 2006;Huettel etal., 2005,^2006^;Volz et al., 2003) have been implicated inthe processing of uncertainty (for an extended discussion of the role of theanterior insula, please see theSupplementary Material). The IPL has previously beenlinked to signalling the moment of expectation violation in magic tricks (Danek et al., 2015), the induction ofcuriosity in a lottery task (van Lieshout etal., 2018), as well as within the trivia question paradigm (Duan etal., 2020;Ligneul et al., 2018), andeven more broadly, to knowledge uncertainty (Volz et al., 2004). Likewise, the IFG has previously been implicatedin the elicitation of curiosity within the trivia question paradigm (Gruber et al., 2014;Kang et al., 2009). According to the Prediction,Appraisal, Curiosity, and Exploration (PACE) framework explaining how curiosityenhances HPC-dependent memory (Gruber &Ranganath, 2019), the IFG plays a critical role in appraisalprocesses. It is involved in determining whether prediction errors andassociated uncertainty elicit curiosity or anxiety. Furthermore, the IFG hasbeen linked to the violation of expectations (Danek et al., 2015) and causal relationships (Parris et al., 2009) in magic tricks. This suggeststhat as participants watch magic tricks, the curiosity IS-RSA effects reportedhere could reflect uncertainty-related signals and their appraisal processes.Specifically, these processes in response to experienced prediction errors mightshare a similar signature when individuals share a sense of curiosity.

Second, significant CMLE effects were observed in broad cortical areas but,importantly, these effects were mostly negative. Indeed, negative clusters werefound across largely distributed cortical and subcortical areas, including majorparts of the DMN (e.g., bilateral ACC, angular gyrus, middle temporal gyrus),FPN (e.g., bilateral MFG, SMA), dorsal attention network (e.g., bilateralposterior superior parietal lobe), ventral attention network (e.g., anteriorinsula/frontal operculum (aI/fO)), as well as visual network. A recentre-analysis of the dataset fromGruber andcolleagues (2014)showed that the DMN and a subnetwork within the FPN(i.e., lateral PFC, posterior inferior temporal gyrus, and superior parietallobe) show a curiosity-by-memory interaction (Murphy, Ranganath, et al., 2021). Our findings not only replicatebut also expand on these results. In alignment with the ROI analysis discussedabove, we found that participants with higher CMLE scores, compared to thosewith lower scores, demonstrated more individualised and variable activation inthese brain networks (for a discussion of the implications of these findings,please refer to theSupplementary Material).

### 4.4 Overall conclusion

Our study demonstrated that the curiosity effect of memory can be replicatedusing naturalistic stimuli. Using analysis approaches to account for the dynamicnature of the magic trick stimuli, we discovered that the effects of curiosityand incentives were not confined to the reward network in the brain per se, butdistributed across various cortical areas. While effects of memory and CMLE werefound within the hippocampal-VTA loop, they also appeared in widespread corticalclusters. This finding challenges the traditional focus on mesolimbic structuresalone, often identified in studies using more reductionist, simple stimuli thatmay not accurately reflect real-life perception and cognition. Additionally,these studies typically rely on less rigorous modelling of the hemodynamicresponse, which may not capture the full complexity of brain function. Ourresults suggest that a stringent focus on specific ROIs could lead to anoversimplified understanding of how the brain processes and encodes naturalisticstimuli. To derive a better understanding on how curiosity influences memory,more research with various stimuli and tasks is needed. Such studies couldprovide invaluable insights for practitioners in educational settings, helpingtailor learning approaches that more effectively align with how the brainnaturally processes information.

## Data and Code Availability

The whole MRI dataset has been made publicly available as the Magic, Memory, andCuriosity (MMC) Dataset (https://doi.org/10.18112/openneuro.ds004182.v1.0.0). The code isavailable on Github (https://github.com/stefaniemeliss/MIL_paper), and Unthresholdedstatistical maps were uploaded to NeuroVault (https://neurovault.org/collections/12980/).

## Author Contributions

Stef Meliss: Conceptualisation, Methodology, Validation, Formal Analysis,Investigation, Writing—Original Draft, Visualisation, and Projectadministration. Aki Tsuchiyagaito: Formal Analysis, Writing—Review &Editing. Phoenix Byrne: Validation, Writing—Review & Editing. Carienvan Reekum: Supervision, Writing—Review & Editing. Kou Murayama:Conceptualisation, Methodology, Supervision, Writing—Review & Editing,and Funding Acquisition.

## Funding

This research was supported by Leverhulme Trust Research Leadership Award(RL-2016-030), Jacobs Foundation Advanced Research Fellowship, and the Alexander vonHumboldt Foundation (the Alexander von Humboldt Professorship endowed by the GermanFederal Ministry of Education and Research) awarded to Kou Murayama.

## Declaration of Competing Interest

We have no conflicts of interest.

## Supplementary Material

Supplementary Material

## Footnotes

1 Another difference between trivia questions and magic tricks is that peopleusually do not expect to see the resolution of curiosity in magic tricks (i.e.,how the magic trick was done), whereas answers are typically presented in triviaquestion paradigm. However, previous neuroimaging work also suggested that theeffect of resolution expectation is not that big (Ligneul et al., 2018).

2 In previous literature on motivated learning, the terms ‘rewards’and ‘incentives’ have been used rather interchangeably (e.g.,Adcock et al., 2006), but some attachdistinct definitions to them. Specifically, incentives are ‘plans thathave predetermined criteria and standards, as well as understood policies fordetermining and allocating rewards’ (Greene, 2010, p. 219). As such, incentives can be seen as a promiseof later rewards, hence incentives can be seen as expected rewards (Berridge, 2000), whereas rewards are theoutcome of motivated behaviour that are received/perceived/consumed (Matyjek et al., 2020). In this paper, weadopt these differential definitions.

3 Due to an error, one item was not included into the inventory. The pressure scalewas computed based on 4 instead of 5 items.

4 50% additional bonus payment should have translated to £0.40 per correctanswer. However, no participant reported to have noticed this error.

5 Due to singular fit warnings for the dependent variable high confidencerecognition in the fMRI data, the model was also executed using a simplifiedrandom effects structure where the random intercepts of subject and randomslopes of subjects for the curiosity effect were specified, but randomintercepts of stimuli were removed allowing the model to converge withoutwarnings. The individual curiosity beta values from both models were highlycorrelated (r = 0.992) and the gLME model specification did not affectthe IS-RSA whole-brain results (correlation unthresholded effect size map= .996, correlation unthresholded statistics map = .997, dicecoefficient of masked cluster-extent thresholded results = 0.960) norreported ROI results.
